# Poly[[(μ_3_-adamantane-1,3-di­acetato)[μ-*N*-(pyridin-3-yl)isonicotinamide]­nickel(II)] monohydrate], a layered coordination polymer with triangular (3,6) topology

**DOI:** 10.1107/S2414314623008696

**Published:** 2023-10-05

**Authors:** Jamelah Z. Travis, Robert L. LaDuca

**Affiliations:** a Department of Chemistry, Hope College, Holland, MI 49423, USA; b E-194 Holmes Hall, Michigan State University, Lyman Briggs College, 919 E. Shaw Lane, East Lansing, MI 48825, USA; Benemérita Universidad Autónoma de Puebla, México

**Keywords:** crystal structure, polymer, topology, nickel, isonicotinamide

## Abstract

The title compound, {[Ni(C_14_H_18_O_4_)(C_11_H_9_N_3_O)]·H_2_O}_*n*_, contains octa­hedrally coordinated Ni^II^ ions ligated by 1,3-adamantanedi­acetato (ada) ligands and *N*-(pyridin-3-yl)isonicotinamide (3-pina) ligands, to form coordination polymer layers with a triangular (3,6) grid topology based on [Ni_2_(OCO)_2_] dimeric units. The diperiodic layer motifs stack in an *ABAB* pattern mediated by C—H⋯O supra­molecular inter­actions between ada ligands and water mol­ecules of crystallization to form the full triperiodic crystal structure of the title compound.

## Abstract 2

The title inorganic Ni^II^ polymer features layers with a triangular (3,6) grid topology, stacked in a *ABAB* pattern to form a triperiodic structure.

## Graphical abstract

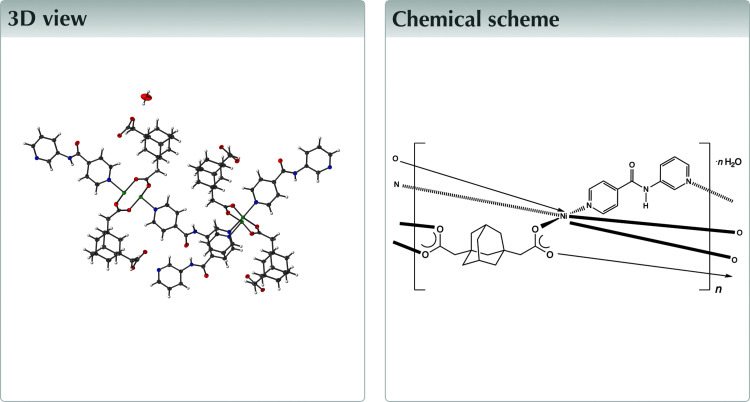

## Structure description

The title complex was obtained during attempts to prepare divalent nickel coordination polymers featuring 1,3-adamantanedi­acetate (ada) ligands and the hydrogen-bonding-capable di­pyridyl­amide ligand *N*-(pyridin-3-yl)isonicotinamide (3-pina). We have reported nickel ada coordination polymers featuring *N*,*N*′-(ethane-1,2-di­yl)diisonicotinamide (edin) and *N*,*N*′-(propane-1,3-di­yl)diisonicotinamide (pdin) (Travis *et al.*, 2018 ▸). [Ni(ada)(edin)]_*n*_ manifests an intriguing self-penetrated layer structure with a 3,5-connected binodal (4^2^6)(4^2^6^7^8) topology. {[Ni_5_(ada)_5_(pdin)_5_(H_2_O)_5_]·8H_2_O}_*n*_ shows a looped layer structure with a 3-connected (4)(4.8^5^) topology. Additionally, our group reported a cadmium adamantanedi­carboxyl­ate (adc) coordination polymer containing 3-pina coligands (LaRose & LaDuca, 2017 ▸). The triperiodic phase {[Cd_2_(adc)_2_(3-pina)_2_]·H_2_O}_*n*_ exhibited a non-inter­penetrated network with 6^5^8 **cds** topology.

**Figure 1:**
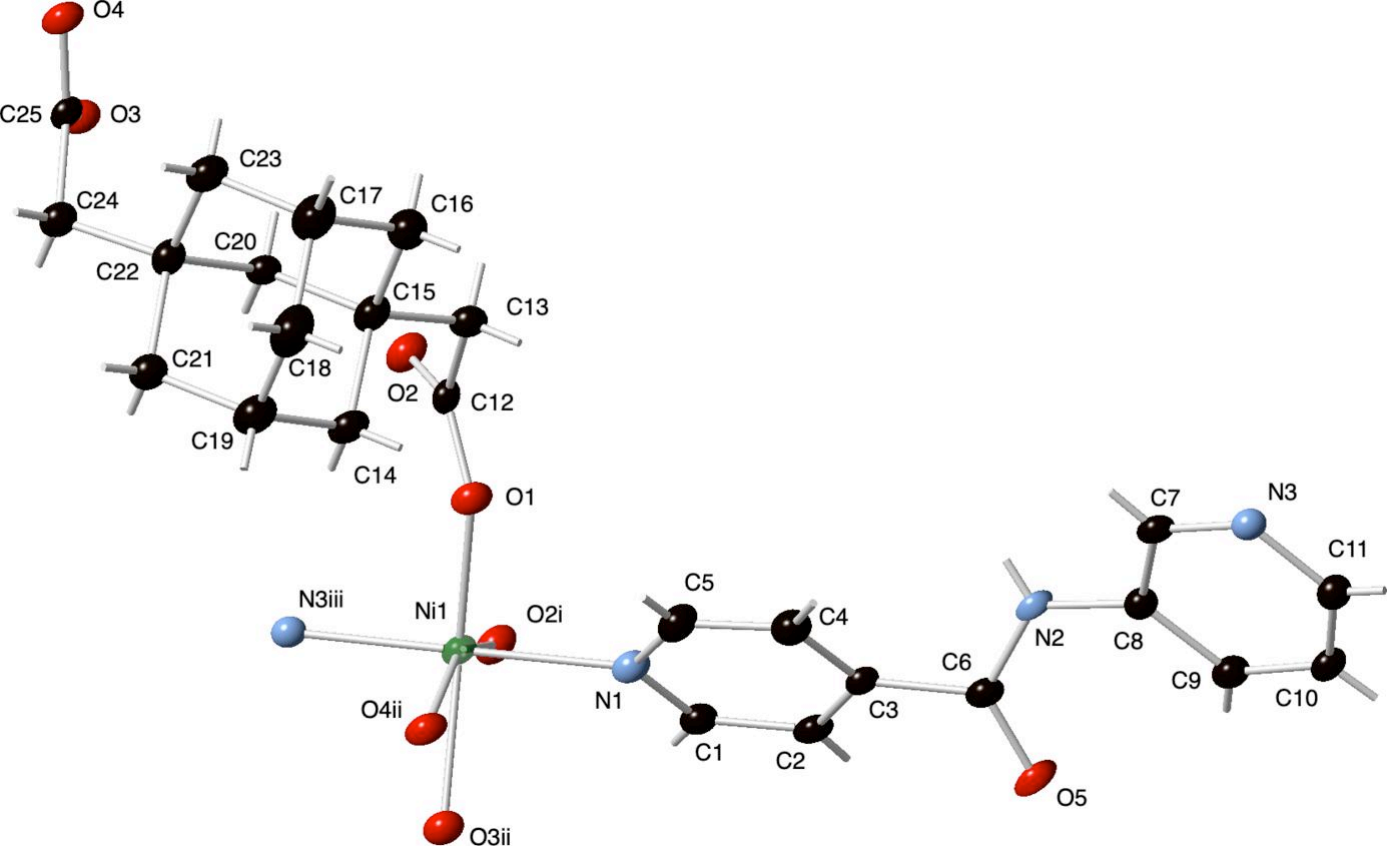
Nickel coordination environment in the title compound with full ligand set. Displacement ellipsoids are drawn at the 50% probability level. Color code: Ni, green; O, red; N, light blue; C, black. H-atom positions are shown as gray sticks. Symmetry codes are as listed in Table 1 ▸.

The asymmetric unit of the title compound contains a nickel atom, a fully deproton­ated ada ligand, an *N*-(pyridin-3-yl)isonicotinamide (3-pina) ligand, and one water mol­ecule of crystallization (Fig. 1 ▸). The Ni atoms possess an octa­hedral {N_2_O_4_} coord­in­ation environment with the nominal axial positions taken up by pyridyl N atom belonging to the isonicotinamide side of a 3-pina ligand, and a pyridyl N atom belonging to the 3-pyridyl side of another 3-pina ligand. The nominal equatorial plane contains a chelating carboxyl­ate group from an ada ligand, and *cis*-oriented O atom donors belonging to two different ada ligands. Bond lengths and angles within the coordination environment are listed in Table 1 ▸.

**Figure 2:**
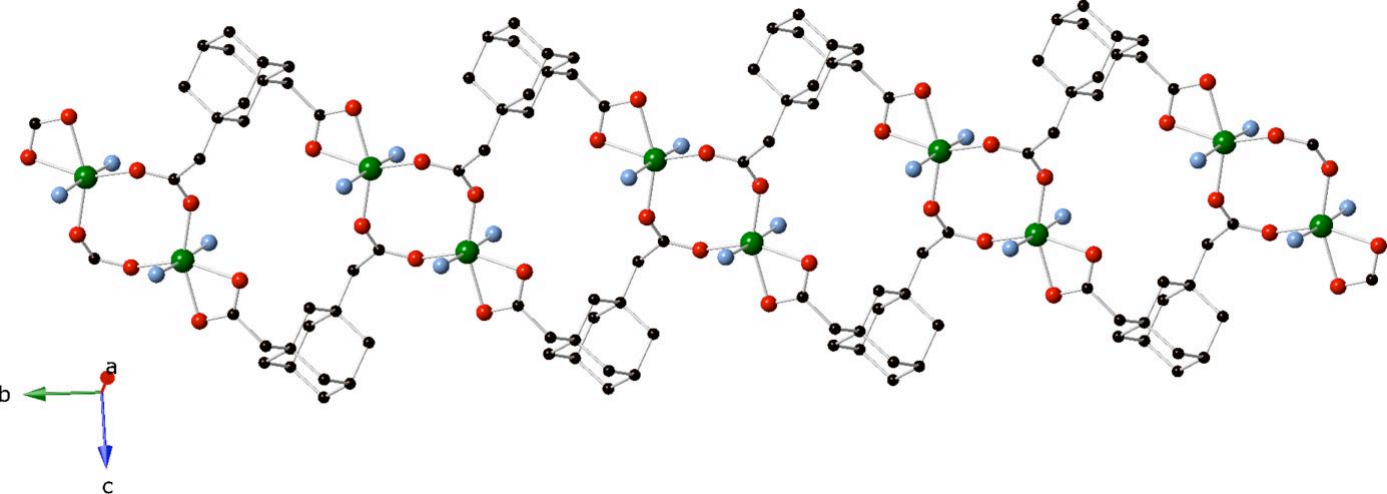
[Ni(ada)]_*n*_ coordination polymer chain motif in the title compound, featuring [Ni_2_(OCO)_2_] dimeric units.

**Figure 3:**
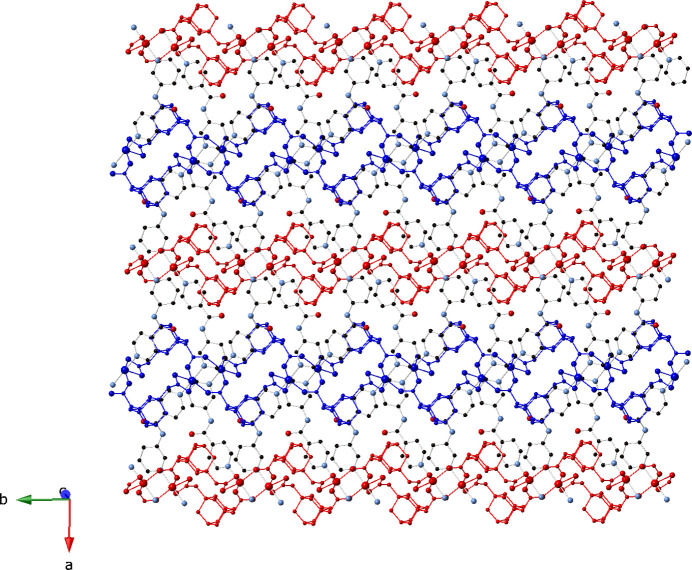
[Ni(ada)(3-pina)]_*n*_ coordination polymer layer motif in the title compound.

**Figure 4:**
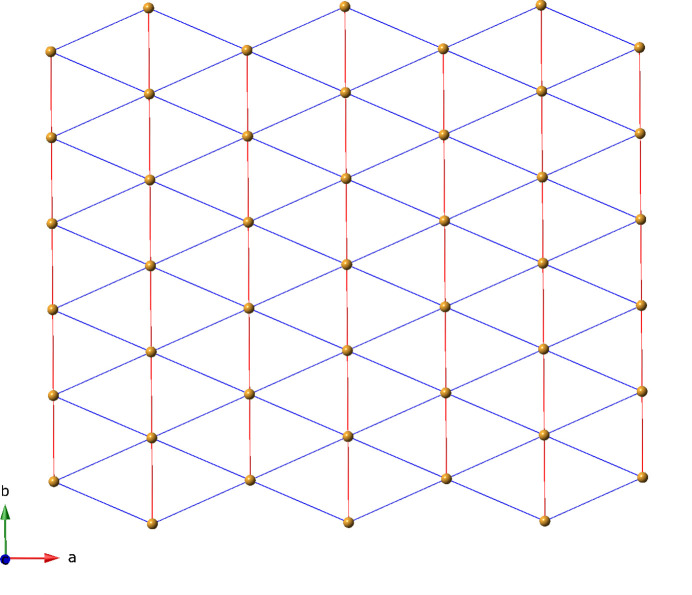
Schematic perspective of the 6-connected (3,6) triangular layer topology in the title compound. Centroids of the [Ni_2_(OCO)_2_] dimeric units are shown as gold spheres. Connections mediated by the ada ligands and 3-pina ligands are drawn as red rods and blue rods, respectively.

The bridging/chelating ada ligands connect to three Ni atoms, and form [Ni(ada)]_*n*_ monoperiodic coordination polymer chains arranged along the *b*-axis direction (Fig. 2 ▸). The chain motifs contain embedded *syn–syn* bridged [Ni_2_(OCO)_2_] dimeric units with an Ni⋯Ni through-space distance of 4.277 (1) Å. Adjacent and parallel chain motifs are pillared by 3-pina ligands into diperiodic coordination polymer layers of stoichiometry [Ni(ada)(3-pina)]_*n*_ (Fig. 3 ▸); these are oriented parallel to the *ab* crystal planes. The topology of the title compound can be inferred by considering the [Ni_2_(OCO)_2_] dimeric units as 6-connected nodes, with two connections provided by the full span of the ada ligands. Each [Ni_2_(OCO)_2_] dimeric unit node also connects to four others *via* 3-pina ligands. The resultant 6-connected layered topology is that of a (3,6) triangular net (Fig. 4 ▸).

**Figure 5:**
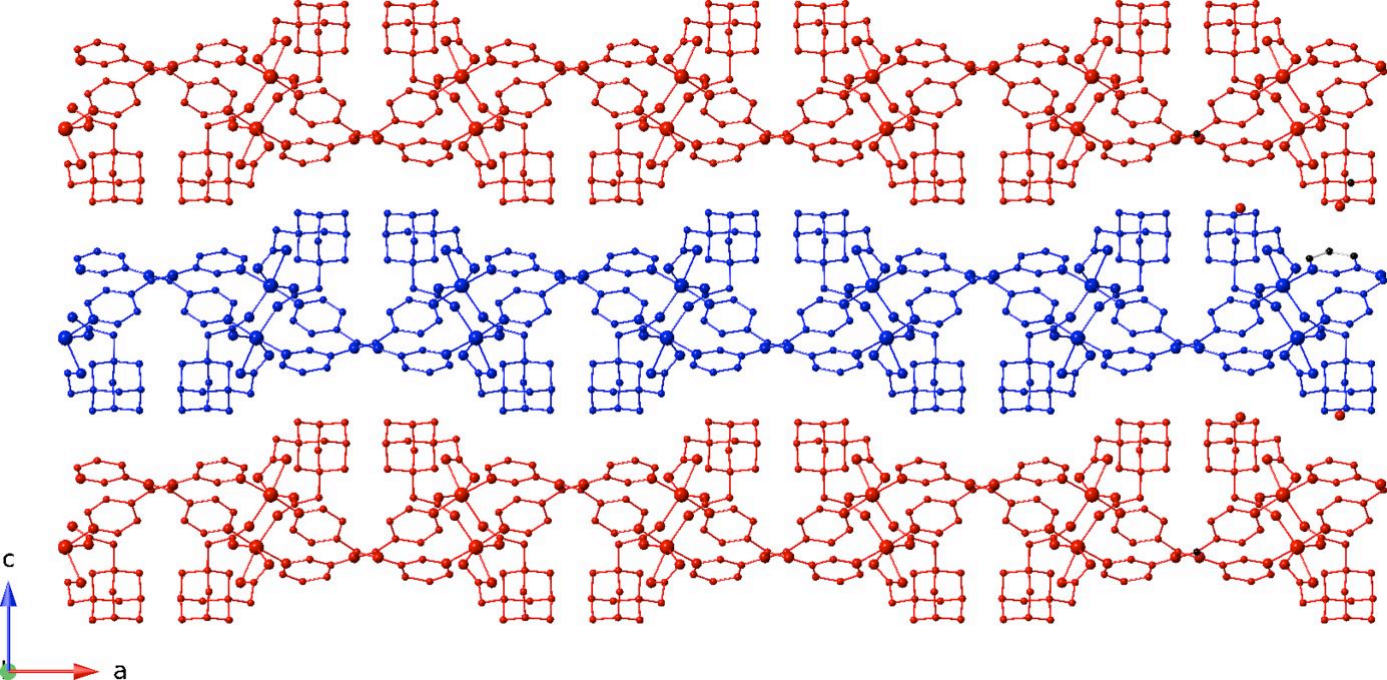
*ABAB* stacking of coordination polymer layers in the title compound.

Parallel [Ni(ada)(3-pina)]_*n*_ layer motifs stack in an *ABAB* pattern along the *c*-axis axis, *via* classical and non-classical hydrogen-bonding pathways (Fig. 5 ▸). The water mol­ecules of crystallization are anchored to the layer motifs *via* O—H⋯O hydrogen bonding donation to ada carboxyl­ate O atoms. The water mol­ecules of crystallization engage in inter­lamellar C—H⋯O inter­actions with 3-pina pyridyl C atoms [C⋯O distance = 3.187 (1) Å]. Metrical parameters for the hydrogen bonding in the title compound are given in Table 2 ▸.

## Synthesis and crystallization

Ni(NO_3_)_2_·6H_2_O (108 mg, 0.37 mmol), 1,3-adamantanedi­acetic acid (adaH_2_, 93 mg, 0.37 mmol), *N*-(pyridin-3-yl)isonicotinamide (3-pina, 74 mg, 0.37 mmol), and 0.75 ml of a 1.0 *M* NaOH solution were placed into 10 ml distilled H_2_O in a Teflon-lined acid digestion bomb. The bomb was sealed and heated in an oven at 393 K for 48 h, and then cooled slowly to 273 K. Green crystals of the title complex were obtained in 43% yield.

## Refinement

Crystal data, data collection and structure refinement details are summarized in Table 3 ▸.

## Supplementary Material

Crystal structure: contains datablock(s) I, global. DOI: 10.1107/S2414314623008696/bh4079sup1.cif

Structure factors: contains datablock(s) I. DOI: 10.1107/S2414314623008696/bh4079Isup2.hkl

CCDC reference: 2299081

Additional supporting information:  crystallographic information; 3D view; checkCIF report

## Figures and Tables

**Table 1:** Selected geometric parameters (Å, °)

Ni1—O1	2.023 (2)	Ni1—O4^ii^	2.132 (2)
Ni1—O2^i^	2.038 (2)	Ni1—N1	2.089 (3)
Ni1—O3^ii^	2.131 (2)	Ni1—N3^iii^	2.099 (3)
			
O1—Ni1—O2^i^	112.60 (10)	O2^i^—Ni1—N3^iii^	89.11 (10)
O1—Ni1—O3^ii^	152.96 (9)	O3^ii^—Ni1—O4^ii^	61.82 (9)
O1—Ni1—O4^ii^	91.42 (9)	N1—Ni1—O3^ii^	89.29 (10)
O1—Ni1—N1	86.69 (10)	N1—Ni1—O4^ii^	89.94 (10)
O1—Ni1—N3^iii^	94.55 (10)	N1—Ni1—N3^iii^	176.07 (11)
O2^i^—Ni1—O3^ii^	93.85 (10)	N3^iii^—Ni1—O3^ii^	91.27 (10)
O2^i^—Ni1—O4^ii^	155.53 (9)	N3^iii^—Ni1—O4^ii^	93.75 (10)
O2^i^—Ni1—N1	86.97 (11)		

Symmetry codes: (i) 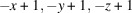 ; (ii) 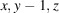 ; (iii) 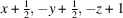 .

**Table 2:** Hydrogen-bond geometry (Å, °)

*D*—H⋯*A*	*D*—H	H⋯*A*	*D*⋯*A*	*D*—H⋯*A*
O1*W*—H1*WA*⋯O4	0.87	1.98	2.809 (5)	159
N2—H2⋯O5^iv^	0.88	2.00	2.874 (4)	173
C1—H1⋯O2^i^	0.95	2.49	2.957 (4)	111
C5—H5⋯O1	0.95	2.48	2.928 (4)	109
C7—H7⋯O2^v^	0.95	2.68	3.027 (4)	102
C9—H9⋯O5	0.95	2.41	2.922 (4)	114

Symmetry codes: (i) 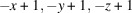 ; (iv) 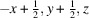 ; (v) 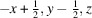 .

**Table 3:** Experimental details

Crystal data	
Chemical formula	[Ni(C_14_H_18_O_4_)(C_11_H_9_N_3_O)]·H_2_O
*M*_r_	526.22
Crystal system, space group	Orthorhombic, *P* *b* *c* *a*
Temperature (K)	173
*a*, *b*, *c* (Å)	21.789 (3), 9.5494 (12), 22.200 (3)
*V* (Å^3^)	4619.2 (10)
*Z*	8
Radiation type	Mo *K*α
μ (mm^−1^)	0.89
Crystal size (mm)	0.30 × 0.08 × 0.05

Data collection	
Diffractometer	Bruker APEXII CCD
Absorption correction	Multi-scan (*SADABS*; Krause *et al.*, 2015 ▸)
*T*_min_, *T*_max_	0.606, 0.745
No. of measured, independent and observed [*I* > 2σ(*I*)] reflections	71364, 4216, 3175
*R*_int_	0.112
(sin θ/λ)_max_ (Å^−1^)	0.602

Refinement	
*R*[*F* ^2^ > 2σ(*F* ^2^)], *wR*(*F* ^2^), *S*	0.048, 0.134, 1.05
No. of reflections	4216
No. of parameters	319
No. of restraints	1
H-atom treatment	H-atom parameters constrained
Δρ_max_, Δρ_min_ (e Å^−3^)	0.76, −0.45

Computer programs: *COSMO* (Bruker, 2009 ▸), *SAINT* (Bruker, 2014 ▸), *SHELXT2018/2* (Sheldrick, 2015*a* ▸), *SHELXL2018/3* (Sheldrick, 2015*b* ▸), *CrystalMaker X* (Palmer, 2020 ▸), and *OLEX2* (Dolomanov *et al.*, 2009 ▸).

## full crystallographic data

### Crystal data

**Table:**

[Ni(C_14_H_18_O_4_)(C_11_H_9_N_3_O)]·H_2_O	*D*_x_ = 1.513 Mg m^−^^3^
*M**_r_* = 526.22	Mo *K*α radiation, λ = 0.71073 Å
Orthorhombic, *P**b**c**a*	Cell parameters from 9950 reflections
*a* = 21.789 (3) Å	θ = 2.5–25.2°
*b* = 9.5494 (12) Å	µ = 0.89 mm^−^^1^
*c* = 22.200 (3) Å	*T* = 173 K
*V* = 4619.2 (10) Å^3^	Needle, green
*Z* = 8	0.30 × 0.08 × 0.05 mm
*F*(000) = 2208	

### Data collection

**Table:**

Bruker APEXII CCD diffractometer	4216 independent reflections
Radiation source: sealed tube	3175 reflections with *I* > 2σ(*I*)
Graphite monochromator	*R*_int_ = 0.112
Detector resolution: 836.6 pixels mm^-1^	θ_max_ = 25.3°, θ_min_ = 1.8°
φ and ω scans	*h* = −26→26
Absorption correction: multi-scan (SADABS; Krause *et al.*, 2015)	*k* = −11→11
*T*_min_ = 0.606, *T*_max_ = 0.745	*l* = −26→26
71364 measured reflections	

### Refinement

**Table:**

Refinement on *F*^2^	Primary atom site location: structure-invariant direct methods
Least-squares matrix: full	Secondary atom site location: difference Fourier map
*R*[*F*^2^ > 2σ(*F*^2^)] = 0.048	Hydrogen site location: mixed
*wR*(*F*^2^) = 0.134	H-atom parameters constrained
*S* = 1.05	*w* = 1/[σ^2^(*F*_o_^2^) + (0.0727*P*)^2^ + 4.9019*P*] where *P* = (*F*_o_^2^ + 2*F*_c_^2^)/3
4216 reflections	(Δ/σ)_max_ = 0.001
319 parameters	Δρ_max_ = 0.76 e Å^−^^3^
1 restraint	Δρ_min_ = −0.44 e Å^−^^3^

### Special details

**Table:**

Refinement. All H atoms attached to C and N atoms were placed in calculated positions and refined with a riding model. The H atoms in the water molecule of crystallization could not be found in a difference map, so they were placed in calculated positions.

### Fractional atomic coordinates and isotropic or equivalent isotropic displacement parameters (Å^2^)

**Table:**

	*x*	*y*	*z*	*U*_iso_*/*U*_eq_	
Ni1	0.48258 (2)	0.33446 (4)	0.56257 (2)	0.01734 (16)	
O1	0.42593 (10)	0.5016 (2)	0.55617 (11)	0.0231 (5)	
O1W	0.3792 (3)	1.3710 (4)	0.74898 (18)	0.0821 (13)	
H1WA	0.390684	1.346055	0.713002	0.123*	
H1WB	0.360617	1.450646	0.743741	0.123*	
O2	0.46532 (11)	0.6932 (2)	0.51276 (11)	0.0234 (6)	
O3	0.51363 (11)	1.1459 (2)	0.60389 (11)	0.0220 (5)	
O4	0.44420 (11)	1.2773 (2)	0.64741 (11)	0.0221 (6)	
O5	0.27987 (11)	−0.1308 (2)	0.41445 (13)	0.0302 (6)	
N1	0.41408 (12)	0.2205 (3)	0.51861 (13)	0.0201 (6)	
N2	0.22128 (13)	0.0684 (3)	0.41194 (13)	0.0217 (7)	
H2	0.224054	0.160150	0.414096	0.026*	
N3	0.05497 (12)	0.0519 (3)	0.39871 (12)	0.0178 (6)	
C1	0.42734 (15)	0.1183 (3)	0.47886 (16)	0.0203 (8)	
H1	0.469232	0.096548	0.471564	0.024*	
C2	0.38285 (16)	0.0435 (3)	0.44816 (15)	0.0202 (8)	
H2A	0.394093	−0.029367	0.421168	0.024*	
C3	0.32140 (15)	0.0766 (3)	0.45745 (15)	0.0182 (7)	
C4	0.30765 (15)	0.1819 (4)	0.49923 (16)	0.0220 (8)	
H4	0.266192	0.206588	0.507133	0.026*	
C5	0.35474 (16)	0.2495 (4)	0.52878 (16)	0.0231 (8)	
H5	0.344752	0.319468	0.557552	0.028*	
C6	0.27282 (15)	−0.0048 (4)	0.42567 (15)	0.0196 (8)	
C7	0.11246 (16)	0.0945 (3)	0.40809 (15)	0.0191 (7)	
H7	0.118697	0.184913	0.424881	0.023*	
C8	0.16377 (15)	0.0138 (3)	0.39462 (15)	0.0181 (7)	
C9	0.15514 (16)	−0.1157 (4)	0.36646 (16)	0.0205 (8)	
H9	0.189018	−0.172723	0.355280	0.025*	
C10	0.09520 (16)	−0.1579 (3)	0.35549 (16)	0.0209 (8)	
H10	0.087462	−0.244906	0.336192	0.025*	
C11	0.04719 (16)	−0.0741 (3)	0.37246 (15)	0.0208 (8)	
H11	0.006559	−0.106242	0.365398	0.025*	
C12	0.42397 (15)	0.6283 (3)	0.54030 (15)	0.0178 (7)	
C13	0.36516 (16)	0.7067 (4)	0.55572 (16)	0.0212 (8)	
H13A	0.330417	0.640170	0.553238	0.025*	
H13B	0.358407	0.779695	0.524739	0.025*	
C14	0.36697 (17)	0.6684 (3)	0.66913 (16)	0.0229 (8)	
H14A	0.406004	0.615760	0.666328	0.027*	
H14B	0.332713	0.601025	0.665009	0.027*	
C15	0.36373 (16)	0.7771 (4)	0.61809 (16)	0.0206 (8)	
C16	0.30221 (16)	0.8574 (4)	0.62426 (17)	0.0243 (8)	
H16A	0.298905	0.927903	0.591727	0.029*	
H16B	0.267546	0.791093	0.620060	0.029*	
C17	0.29848 (17)	0.9305 (4)	0.68564 (17)	0.0282 (9)	
H17	0.258643	0.982035	0.688746	0.034*	
C18	0.30197 (18)	0.8208 (4)	0.73539 (18)	0.0314 (9)	
H18A	0.267426	0.754089	0.731347	0.038*	
H18B	0.298775	0.866780	0.775231	0.038*	
C19	0.36298 (17)	0.7421 (4)	0.73080 (16)	0.0261 (8)	
H19	0.365533	0.670741	0.763675	0.031*	
C20	0.41681 (15)	0.8826 (3)	0.62541 (15)	0.0174 (7)	
H20A	0.456463	0.832699	0.621716	0.021*	
H20B	0.414628	0.953113	0.592798	0.021*	
C21	0.41646 (17)	0.8455 (4)	0.73669 (16)	0.0242 (8)	
H21A	0.455797	0.793969	0.734058	0.029*	
H21B	0.414621	0.891740	0.776565	0.029*	
C22	0.41396 (16)	0.9572 (4)	0.68684 (15)	0.0208 (8)	
C23	0.35179 (17)	1.0350 (4)	0.69142 (18)	0.0254 (8)	
H23A	0.349127	1.083998	0.730630	0.030*	
H23B	0.348930	1.105950	0.659028	0.030*	
C24	0.46870 (17)	1.0572 (4)	0.69556 (16)	0.0231 (8)	
H24A	0.463732	1.106082	0.734587	0.028*	
H24B	0.506748	1.000850	0.697935	0.028*	
C25	0.47650 (16)	1.1662 (3)	0.64650 (16)	0.0199 (8)	

### Atomic displacement parameters (Å^2^)

**Table:**

	*U*^11^	*U*^22^	*U*^33^	*U*^12^	*U*^13^	*U*^23^
Ni1	0.0174 (2)	0.0116 (2)	0.0231 (3)	−0.00055 (17)	0.00082 (18)	−0.00025 (18)
O1	0.0210 (13)	0.0152 (12)	0.0332 (14)	0.0007 (10)	0.0007 (11)	0.0018 (11)
O1W	0.140 (4)	0.049 (2)	0.057 (2)	0.026 (3)	0.017 (3)	0.0070 (19)
O2	0.0243 (13)	0.0187 (12)	0.0271 (14)	−0.0046 (10)	0.0064 (11)	−0.0022 (11)
O3	0.0235 (13)	0.0164 (12)	0.0261 (14)	0.0008 (10)	0.0024 (11)	0.0002 (10)
O4	0.0238 (13)	0.0135 (12)	0.0291 (14)	0.0024 (10)	0.0039 (11)	−0.0009 (10)
O5	0.0239 (14)	0.0104 (12)	0.0563 (18)	−0.0011 (10)	−0.0040 (13)	−0.0058 (12)
N1	0.0178 (15)	0.0145 (14)	0.0280 (16)	−0.0001 (12)	0.0012 (13)	0.0004 (12)
N2	0.0205 (16)	0.0085 (14)	0.0361 (18)	−0.0013 (12)	−0.0021 (13)	−0.0017 (12)
N3	0.0186 (15)	0.0148 (14)	0.0201 (15)	−0.0001 (12)	0.0000 (12)	0.0011 (12)
C1	0.0148 (17)	0.0146 (17)	0.031 (2)	0.0020 (13)	−0.0006 (15)	0.0006 (15)
C2	0.0246 (19)	0.0109 (16)	0.0250 (19)	0.0027 (14)	0.0017 (15)	−0.0002 (14)
C3	0.0205 (18)	0.0105 (16)	0.0236 (18)	−0.0014 (14)	−0.0028 (14)	0.0020 (14)
C4	0.0149 (17)	0.0196 (18)	0.031 (2)	0.0015 (14)	0.0023 (15)	0.0004 (15)
C5	0.0248 (19)	0.0170 (18)	0.027 (2)	−0.0007 (15)	0.0038 (15)	−0.0035 (15)
C6	0.0210 (19)	0.0127 (17)	0.0253 (19)	0.0002 (14)	0.0011 (14)	0.0015 (14)
C7	0.0246 (19)	0.0117 (16)	0.0209 (18)	0.0006 (14)	0.0006 (14)	0.0019 (14)
C8	0.0192 (17)	0.0154 (17)	0.0196 (18)	−0.0009 (14)	−0.0009 (14)	−0.0004 (14)
C9	0.0220 (18)	0.0148 (16)	0.0249 (19)	0.0023 (15)	−0.0003 (15)	−0.0021 (15)
C10	0.0257 (19)	0.0133 (17)	0.0236 (19)	−0.0017 (15)	−0.0016 (15)	−0.0019 (14)
C11	0.0206 (18)	0.0182 (18)	0.0235 (18)	−0.0031 (15)	−0.0023 (15)	0.0007 (15)
C12	0.0208 (18)	0.0147 (17)	0.0180 (17)	−0.0019 (14)	−0.0035 (14)	−0.0029 (14)
C13	0.0214 (18)	0.0156 (17)	0.026 (2)	0.0009 (14)	−0.0009 (15)	0.0024 (14)
C14	0.0238 (18)	0.0146 (17)	0.030 (2)	−0.0032 (15)	0.0024 (15)	0.0024 (15)
C15	0.0210 (18)	0.0170 (17)	0.0237 (19)	0.0008 (14)	0.0028 (15)	−0.0037 (15)
C16	0.0171 (18)	0.0238 (19)	0.032 (2)	0.0011 (15)	0.0015 (15)	−0.0018 (16)
C17	0.023 (2)	0.0242 (19)	0.038 (2)	0.0028 (16)	0.0068 (17)	−0.0097 (17)
C18	0.034 (2)	0.031 (2)	0.029 (2)	−0.0110 (18)	0.0106 (18)	−0.0060 (17)
C19	0.035 (2)	0.0202 (19)	0.0235 (19)	−0.0047 (17)	0.0051 (17)	0.0025 (16)
C20	0.0204 (18)	0.0137 (16)	0.0182 (17)	0.0000 (14)	0.0007 (14)	0.0025 (14)
C21	0.033 (2)	0.0191 (18)	0.0208 (19)	−0.0024 (16)	−0.0016 (16)	0.0014 (15)
C22	0.0247 (19)	0.0171 (17)	0.0208 (18)	−0.0039 (15)	−0.0013 (15)	−0.0006 (14)
C23	0.029 (2)	0.0176 (18)	0.030 (2)	0.0024 (16)	0.0016 (16)	−0.0030 (15)
C24	0.030 (2)	0.0171 (17)	0.0223 (19)	−0.0020 (15)	−0.0021 (15)	0.0006 (15)
C25	0.0247 (19)	0.0124 (17)	0.0225 (18)	−0.0054 (15)	−0.0038 (15)	0.0010 (14)

### Geometric parameters (Å, º)

**Table:**

Ni1—O1	2.023 (2)	C11—H11	0.9500
Ni1—O2^i^	2.038 (2)	C12—C13	1.523 (5)
Ni1—O3^ii^	2.131 (2)	C13—H13A	0.9900
Ni1—O4^ii^	2.132 (2)	C13—H13B	0.9900
Ni1—N1	2.089 (3)	C13—C15	1.539 (5)
Ni1—N3^iii^	2.099 (3)	C14—H14A	0.9900
O1—C12	1.261 (4)	C14—H14B	0.9900
O1W—H1WA	0.8700	C14—C15	1.538 (5)
O1W—H1WB	0.8701	C14—C19	1.542 (5)
O2—C12	1.253 (4)	C15—C16	1.551 (5)
O3—C25	1.260 (4)	C15—C20	1.542 (5)
O4—C25	1.273 (4)	C16—H16A	0.9900
O5—C6	1.238 (4)	C16—H16B	0.9900
N1—C1	1.347 (4)	C16—C17	1.533 (5)
N1—C5	1.342 (4)	C17—H17	1.0000
N2—H2	0.8800	C17—C18	1.525 (5)
N2—C6	1.357 (4)	C17—C23	1.536 (5)
N2—C8	1.411 (4)	C18—H18A	0.9900
N3—C7	1.334 (4)	C18—H18B	0.9900
N3—C11	1.347 (4)	C18—C19	1.530 (5)
C1—H1	0.9500	C19—H19	1.0000
C1—C2	1.384 (5)	C19—C21	1.533 (5)
C2—H2A	0.9500	C20—H20A	0.9900
C2—C3	1.391 (5)	C20—H20B	0.9900
C3—C4	1.400 (5)	C20—C22	1.540 (5)
C3—C6	1.491 (5)	C21—H21A	0.9900
C4—H4	0.9500	C21—H21B	0.9900
C4—C5	1.378 (5)	C21—C22	1.538 (5)
C5—H5	0.9500	C22—C23	1.548 (5)
C7—H7	0.9500	C22—C24	1.541 (5)
C7—C8	1.390 (5)	C23—H23A	0.9900
C8—C9	1.399 (5)	C23—H23B	0.9900
C9—H9	0.9500	C24—H24A	0.9900
C9—C10	1.388 (5)	C24—H24B	0.9900
C10—H10	0.9500	C24—C25	1.516 (5)
C10—C11	1.370 (5)		
			
O1—Ni1—O2^i^	112.60 (10)	C15—C14—H14A	109.6
O1—Ni1—O3^ii^	152.96 (9)	C15—C14—H14B	109.6
O1—Ni1—O4^ii^	91.42 (9)	C15—C14—C19	110.1 (3)
O1—Ni1—N1	86.69 (10)	C19—C14—H14A	109.6
O1—Ni1—N3^iii^	94.55 (10)	C19—C14—H14B	109.6
O2^i^—Ni1—O3^ii^	93.85 (10)	C13—C15—C16	108.2 (3)
O2^i^—Ni1—O4^ii^	155.53 (9)	C13—C15—C20	111.4 (3)
O2^i^—Ni1—N1	86.97 (11)	C14—C15—C13	111.6 (3)
O2^i^—Ni1—N3^iii^	89.11 (10)	C14—C15—C16	108.0 (3)
O3^ii^—Ni1—O4^ii^	61.82 (9)	C14—C15—C20	109.2 (3)
N1—Ni1—O3^ii^	89.29 (10)	C20—C15—C16	108.4 (3)
N1—Ni1—O4^ii^	89.94 (10)	C15—C16—H16A	109.6
N1—Ni1—N3^iii^	176.07 (11)	C15—C16—H16B	109.6
N3^iii^—Ni1—O3^ii^	91.27 (10)	H16A—C16—H16B	108.1
N3^iii^—Ni1—O4^ii^	93.75 (10)	C17—C16—C15	110.5 (3)
C12—O1—Ni1	142.9 (2)	C17—C16—H16A	109.6
H1WA—O1W—H1WB	104.5	C17—C16—H16B	109.6
C12—O2—Ni1^i^	137.5 (2)	C16—C17—H17	109.3
C25—O3—Ni1^iv^	89.42 (19)	C16—C17—C23	109.3 (3)
C25—O4—Ni1^iv^	89.0 (2)	C18—C17—C16	109.1 (3)
C1—N1—Ni1	122.0 (2)	C18—C17—H17	109.3
C5—N1—Ni1	120.2 (2)	C18—C17—C23	110.4 (3)
C5—N1—C1	117.8 (3)	C23—C17—H17	109.3
C6—N2—H2	116.3	C17—C18—H18A	109.8
C6—N2—C8	127.3 (3)	C17—C18—H18B	109.8
C8—N2—H2	116.3	C17—C18—C19	109.4 (3)
C7—N3—Ni1^v^	118.9 (2)	H18A—C18—H18B	108.2
C7—N3—C11	117.3 (3)	C19—C18—H18A	109.8
C11—N3—Ni1^v^	123.0 (2)	C19—C18—H18B	109.8
N1—C1—H1	118.4	C14—C19—H19	109.5
N1—C1—C2	123.1 (3)	C18—C19—C14	109.4 (3)
C2—C1—H1	118.4	C18—C19—H19	109.5
C1—C2—H2A	120.5	C18—C19—C21	109.8 (3)
C1—C2—C3	118.9 (3)	C21—C19—C14	109.1 (3)
C3—C2—H2A	120.5	C21—C19—H19	109.5
C2—C3—C4	117.9 (3)	C15—C20—H20A	109.3
C2—C3—C6	119.6 (3)	C15—C20—H20B	109.3
C4—C3—C6	122.4 (3)	H20A—C20—H20B	108.0
C3—C4—H4	120.3	C22—C20—C15	111.4 (3)
C5—C4—C3	119.5 (3)	C22—C20—H20A	109.3
C5—C4—H4	120.3	C22—C20—H20B	109.3
N1—C5—C4	122.7 (3)	C19—C21—H21A	109.4
N1—C5—H5	118.6	C19—C21—H21B	109.4
C4—C5—H5	118.6	C19—C21—C22	111.0 (3)
O5—C6—N2	123.9 (3)	H21A—C21—H21B	108.0
O5—C6—C3	120.9 (3)	C22—C21—H21A	109.4
N2—C6—C3	115.2 (3)	C22—C21—H21B	109.4
N3—C7—H7	118.2	C20—C22—C23	108.4 (3)
N3—C7—C8	123.5 (3)	C20—C22—C24	111.5 (3)
C8—C7—H7	118.2	C21—C22—C20	108.4 (3)
C7—C8—N2	116.8 (3)	C21—C22—C23	108.5 (3)
C7—C8—C9	118.6 (3)	C21—C22—C24	108.2 (3)
C9—C8—N2	124.6 (3)	C24—C22—C23	111.8 (3)
C8—C9—H9	121.3	C17—C23—C22	110.1 (3)
C10—C9—C8	117.5 (3)	C17—C23—H23A	109.6
C10—C9—H9	121.3	C17—C23—H23B	109.6
C9—C10—H10	120.0	C22—C23—H23A	109.6
C11—C10—C9	120.1 (3)	C22—C23—H23B	109.6
C11—C10—H10	120.0	H23A—C23—H23B	108.1
N3—C11—C10	123.0 (3)	C22—C24—H24A	108.5
N3—C11—H11	118.5	C22—C24—H24B	108.5
C10—C11—H11	118.5	H24A—C24—H24B	107.5
O1—C12—C13	115.9 (3)	C25—C24—C22	115.0 (3)
O2—C12—O1	125.9 (3)	C25—C24—H24A	108.5
O2—C12—C13	118.1 (3)	C25—C24—H24B	108.5
C12—C13—H13A	108.4	O3—C25—Ni1^iv^	59.84 (17)
C12—C13—H13B	108.4	O3—C25—O4	119.7 (3)
C12—C13—C15	115.7 (3)	O3—C25—C24	120.4 (3)
H13A—C13—H13B	107.4	O4—C25—Ni1^iv^	59.89 (17)
C15—C13—H13A	108.4	O4—C25—C24	119.9 (3)
C15—C13—H13B	108.4	C24—C25—Ni1^iv^	175.6 (2)
H14A—C14—H14B	108.2		
			
Ni1—O1—C12—O2	9.4 (6)	C12—C13—C15—C14	−64.2 (4)
Ni1—O1—C12—C13	−171.6 (3)	C12—C13—C15—C16	177.1 (3)
Ni1^i^—O2—C12—O1	50.8 (5)	C12—C13—C15—C20	58.0 (4)
Ni1^i^—O2—C12—C13	−128.2 (3)	C13—C15—C16—C17	−179.6 (3)
Ni1^iv^—O3—C25—O4	−3.3 (3)	C13—C15—C20—C22	177.8 (3)
Ni1^iv^—O3—C25—C24	174.9 (3)	C14—C15—C16—C17	59.5 (4)
Ni1^iv^—O4—C25—O3	3.3 (3)	C14—C15—C20—C22	−58.6 (4)
Ni1^iv^—O4—C25—C24	−174.9 (3)	C14—C19—C21—C22	60.2 (4)
Ni1—N1—C1—C2	−178.5 (3)	C15—C14—C19—C18	60.5 (4)
Ni1—N1—C5—C4	177.2 (3)	C15—C14—C19—C21	−59.6 (4)
Ni1^v^—N3—C7—C8	167.3 (3)	C15—C16—C17—C18	−60.6 (4)
Ni1^v^—N3—C11—C10	−169.7 (3)	C15—C16—C17—C23	60.1 (4)
O1—C12—C13—C15	88.5 (4)	C15—C20—C22—C21	58.2 (4)
O2—C12—C13—C15	−92.4 (4)	C15—C20—C22—C23	−59.3 (4)
N1—C1—C2—C3	1.5 (5)	C15—C20—C22—C24	177.2 (3)
N2—C8—C9—C10	176.3 (3)	C16—C15—C20—C22	58.8 (4)
N3—C7—C8—N2	−174.6 (3)	C16—C17—C18—C19	60.6 (4)
N3—C7—C8—C9	3.9 (5)	C16—C17—C23—C22	−60.5 (4)
C1—N1—C5—C4	−1.8 (5)	C17—C18—C19—C14	−60.5 (4)
C1—C2—C3—C4	−2.0 (5)	C17—C18—C19—C21	59.1 (4)
C1—C2—C3—C6	−178.8 (3)	C18—C17—C23—C22	59.6 (4)
C2—C3—C4—C5	0.8 (5)	C18—C19—C21—C22	−59.6 (4)
C2—C3—C6—O5	33.1 (5)	C19—C14—C15—C13	−177.9 (3)
C2—C3—C6—N2	−148.4 (3)	C19—C14—C15—C16	−59.1 (4)
C3—C4—C5—N1	1.2 (5)	C19—C14—C15—C20	58.6 (4)
C4—C3—C6—O5	−143.5 (4)	C19—C21—C22—C20	−59.0 (4)
C4—C3—C6—N2	35.0 (5)	C19—C21—C22—C23	58.4 (4)
C5—N1—C1—C2	0.4 (5)	C19—C21—C22—C24	179.9 (3)
C6—N2—C8—C7	152.4 (3)	C20—C15—C16—C17	−58.7 (4)
C6—N2—C8—C9	−26.1 (5)	C20—C22—C23—C17	59.5 (4)
C6—C3—C4—C5	177.5 (3)	C20—C22—C24—C25	55.4 (4)
C7—N3—C11—C10	0.0 (5)	C21—C22—C23—C17	−58.0 (4)
C7—C8—C9—C10	−2.1 (5)	C21—C22—C24—C25	174.5 (3)
C8—N2—C6—O5	13.6 (6)	C22—C24—C25—O3	−96.9 (4)
C8—N2—C6—C3	−164.8 (3)	C22—C24—C25—O4	81.2 (4)
C8—C9—C10—C11	−0.4 (5)	C23—C17—C18—C19	−59.5 (4)
C9—C10—C11—N3	1.6 (5)	C23—C22—C24—C25	−66.1 (4)
C11—N3—C7—C8	−2.8 (5)	C24—C22—C23—C17	−177.2 (3)

Symmetry codes: (i) −*x*+1, −*y*+1, −*z*+1; (ii) *x*, *y*−1, *z*; (iii) *x*+1/2, −*y*+1/2, −*z*+1; (iv) *x*, *y*+1, *z*; (v) *x*−1/2, −*y*+1/2, −*z*+1.

### Hydrogen-bond geometry (Å, º)

**Table:**

*D*—H···*A*	*D*—H	H···*A*	*D*···*A*	*D*—H···*A*
O1*W*—H1*WA*···O4	0.87	1.98	2.809 (5)	159
N2—H2···O5^vi^	0.88	2.00	2.874 (4)	173
C1—H1···O2^i^	0.95	2.49	2.957 (4)	111
C5—H5···O1	0.95	2.48	2.928 (4)	109
C7—H7···O2^vii^	0.95	2.68	3.027 (4)	102
C9—H9···O5	0.95	2.41	2.922 (4)	114

Symmetry codes: (i) −*x*+1, −*y*+1, −*z*+1; (vi) −*x*+1/2, *y*+1/2, *z*; (vii) −*x*+1/2, *y*−1/2, *z*.
